# Supplementary material for: Highly tunable ground and excited state excitonic dipoles in multilayer 2H-MoSe2
Source: Nat Commun. 2024 May 23;15:4377. doi: 10.1038/s41467-024-48476-x (PMC11519368; doi:10.1038/s41467-024-48476-x)
Supplement: Supplementary file 1 — The new supplementary information [file 41467_2024_48476_MOESM1_ESM.pdf]

Supplemental information for "Highly Tunable Ground and Excited State Excitonic Dipoles in Multilayer 2H-$MoSe_2$"

Shun Feng,[1,*] Aidan Campbell,[1,*] Mauro Brotons-Gisbert,[1,†] Daniel Andres-Penares,[1] Hyeonjun Baek,[1] Takashi Taniguchi,[2] Kenji Watanabe,[3] Bernhard Urbaszek,[4] Iann C. Gerber,[5] and Brian D. Gerardot[1,‡]

[1] *Institute of Photonics and Quantum Sciences, SUPA, Heriot-Watt University, Edinburgh EH14 4AS, UK*
[2] *International Center for Materials Nanoarchitectonics, National Institute for Materials Science, 1-1 Namiki, Tsukuba 305-0044, Japan*
[3] *Research Center for Functional Materials, National Institute for Materials Science, 1-1 Namiki, Tsukuba 305-0044, Japan*
[4] *Institute of Condensed Matter Physics, Technische Universität Darmstadt, 64289 Darmstadt, Germany*
[5] *Université de Toulouse, INSA-CNRS-UPS, LPCNO, 135 Avenue de Rangueil, 31077 Toulouse, France*

(Dated: April 19, 2024)

* These two authors contributed equally
† m.brotons_i_gisbert@hw.ac.uk
‡ B.D.Gerardot@hw.ac.uk

1. SUPPLEMENTARY DATA FIGURES

These supplementary figures Figs. S1-S12 are all referenced in the main manuscript.

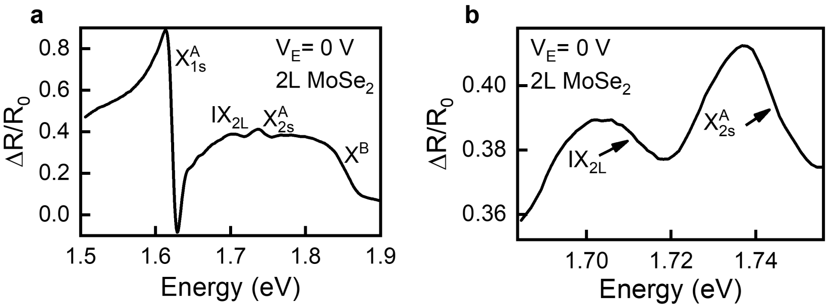

Fig. S1. **Differential reflectance linecuts of the 2H 2L $MoSe_2$ a** Differential reflectance ($\Delta R/R_0$) spectrum for 2H 2L $MoSe_2$ at $V_E = 0$ V. **b** The $\Delta R/R_0$ spectrum in panel (a) shown for a reduced range of energy for clearer visualisation of the interlayer exciton, IX_{2L}, and the A-series $2s$ exciton, X^A_{2s}.

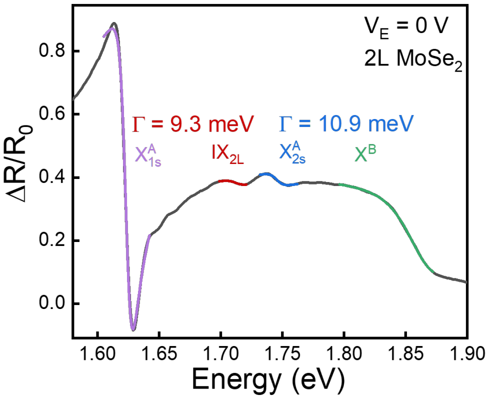

Fig. S2. **Extracting the optical susceptibility of the excitonic states in 2L $MoSe_2$.** The $\Delta R/R_0$ spectrum of 2H 2L $MoSe_2$ at $V_E = 0$ V (black solid line). The coloured solid lines represent fits to the $\Delta R/R_0$ signal for the X^A_{1s}, IX_{2L}, X^A_{2s}, and X_B resonances based on a transfer matrix method. The linewidth Γ of the IX_{2L} and X^A_{2s} transitions is highlighted.

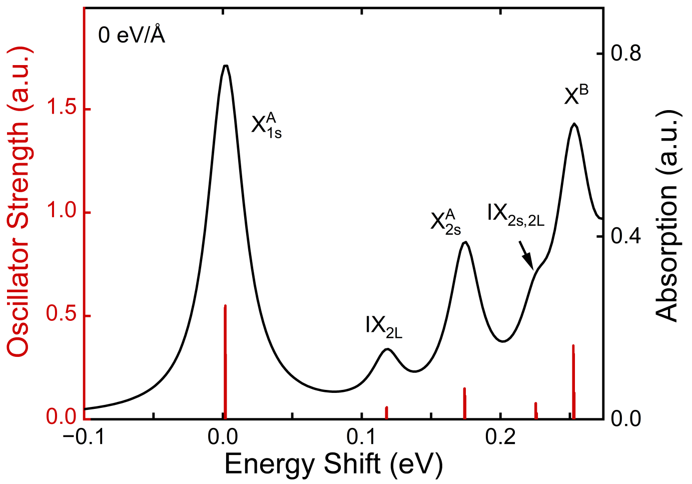

Fig. S3. **Theoretical 2L $MoSe_2$ absorption spectrum at 0 eV/Å electric field** Absorption spectrum (black) and oscillator strength (red) for natural 2H stacking, calculated with the GW + Bethe-Salpeter equation (BSE) method.

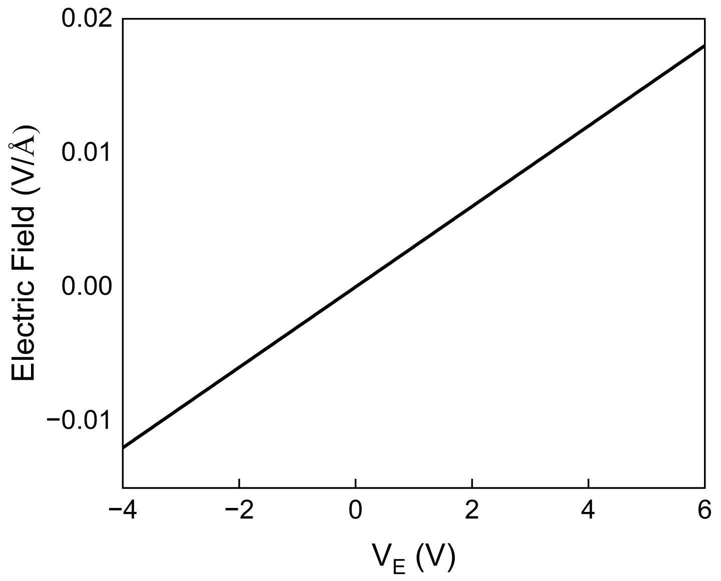

Fig. S4. **Conversion from V_E to electric field to to make it easier for the reader to access.**

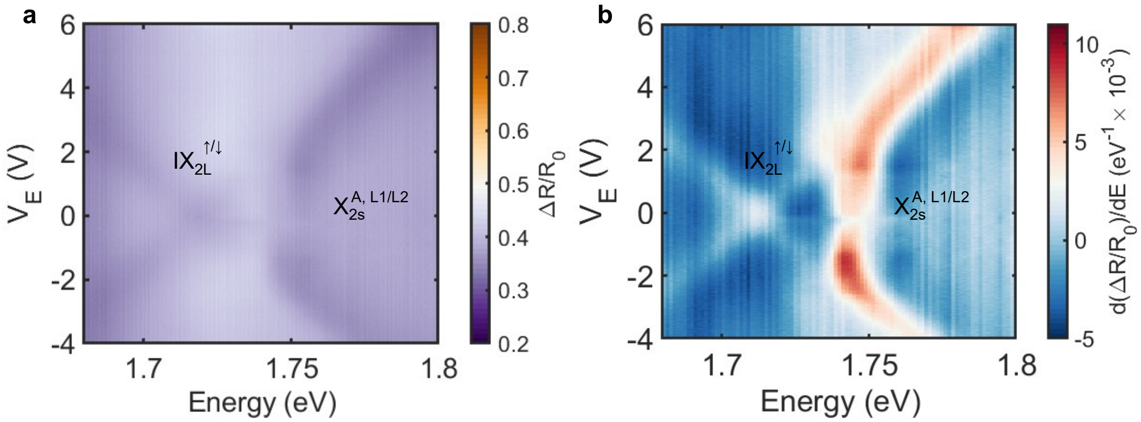

Fig. S5. **Comparison between $\Delta R/R_0$ and d$(\Delta R/R_0)$/dE plots in 2L MoSe$_2$ a** V$_E$ dependent differential reflectance $(\Delta R/R_0)$ in 2L MoSe$_2$. **b** The V$_E$ dependence of the derivative with respect to energy of the the $\Delta R/R_0$ signal in panel (a) (d$(\Delta R/R_0)$/dE). Taking the derivative with respect to energy allows clearer visualisation of the weak excitonic features, IX$_{2L}$ and X$_{2s}^A$, and their hybridisation.

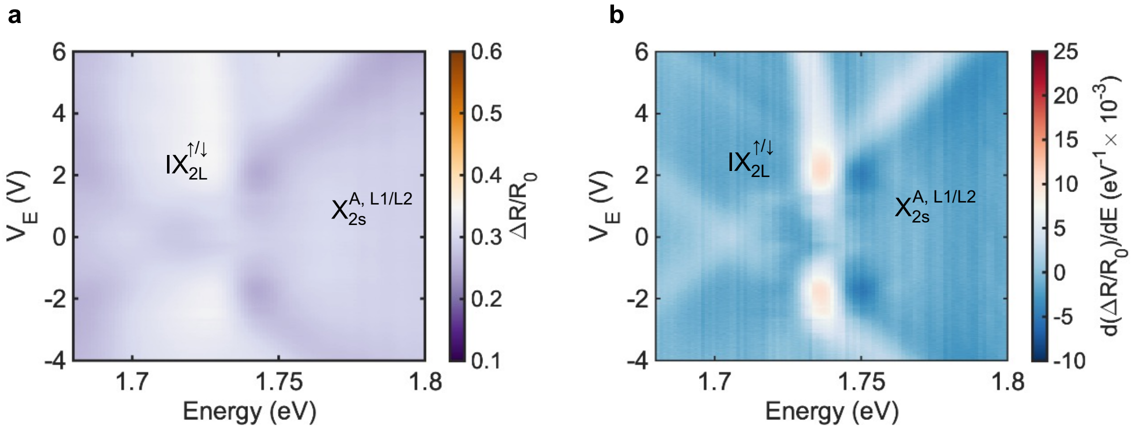

Fig. S6. **Measurements in an alternate 2L MoSe$_2$ location a,b** V$_E$ dependent $\Delta R/R_0$ (a) and d$(\Delta R/R_0)$/dE (b) in a second location in the 2L MoSe$_2$. As previously observed in the main measurement location, we observe a resonance around 1.71 eV which we unambiguously identify as the interlayer exciton, IX$_{2L}$, through its large Stark shift under an applied V$_E$. As this resonance is tuned through $\sim$ 1.74 eV, signatures of hybridisation with the X$_{2s}^A$ are observed.

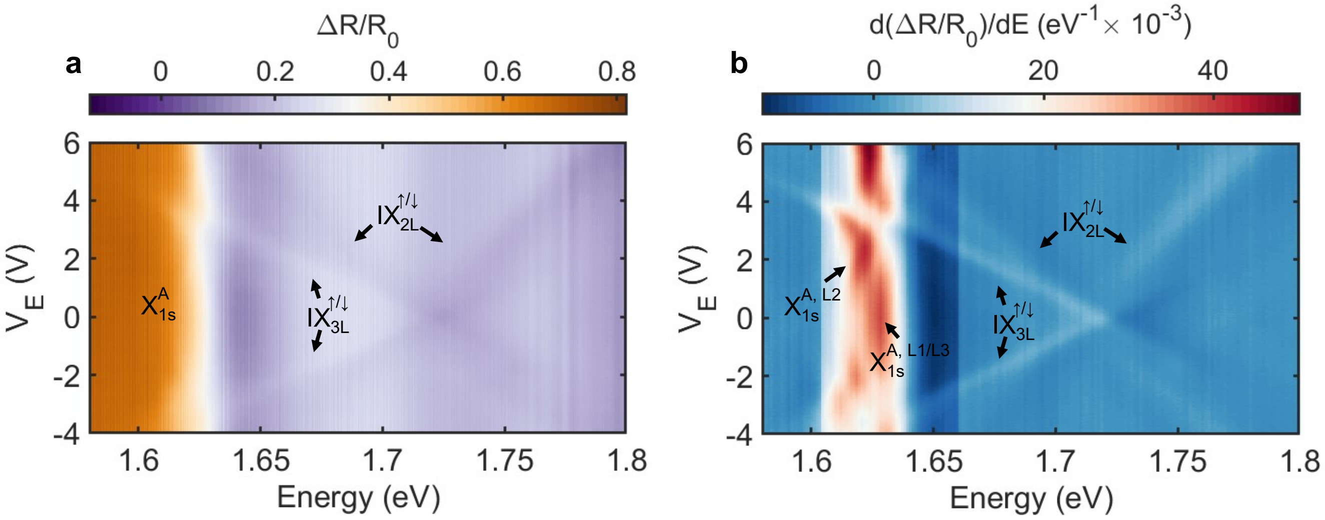

Fig. S7. **Comparison between $\Delta R/R_0$ and d$(\Delta R/R_0)$/dE plots in 3L MoSe$_2$ a** V$_E$ dependent differential reflectance ($\Delta R/R_0$) in 3L MoSe$_2$. b The V$_E$ dependence of the derivative with respect to energy of the $\Delta R/R_0$ signal in panel (a) (d($\Delta R/R_0$)/dE). Taking the derivative with respect to energy allows clearer visualisation of IX$_{3L}$ and its hybridisation with the $X_{1s}^{A,L1/L3}$ excitons.

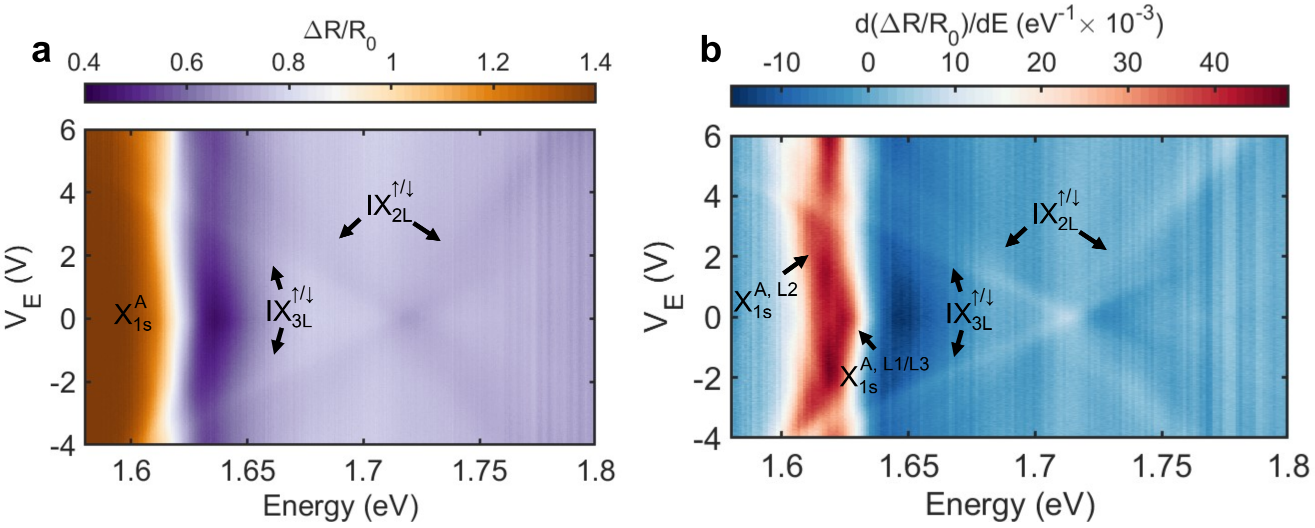

Fig. S8. **Measurements in an alternate 3L MoSe$_2$ location a,b** V$_E$ dependent $\Delta R/R_0$ (a) and d($\Delta R/R_0$)/dE (b) in a second 3L MoSe$_2$ region of our sample. Similar to the main measurement location, at $V_E = 0$ V we observe two resonances around 1.72 eV which we identify as IX$_{3L}$ and IX$_{2L}$ through their distinct electric dipole moments. As also seen in the main measurement location we observe two exciton transitions in the energy range corresponding to X_{1s}^{A} due to the different average permittivity surrounding excitons localised in L2, compared to L1 and L3 of the trilayer. As the energy of IX$_{3L}$ is tuned towards $X_{1s}^{A,L1/L3}$ with V$_E$, there is a clear hybridisation between the features.

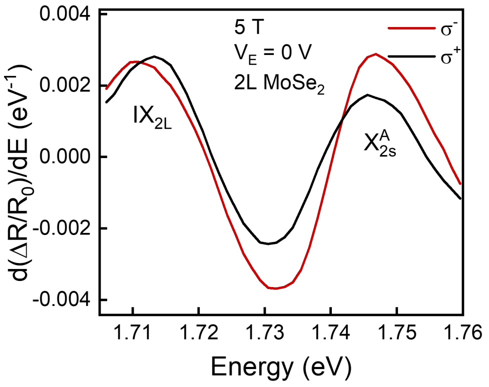

Fig. S9. **Linecuts of the reflection contrast for an applied magnetic field.** σ^+ (black) and σ^- (red) polarisation resolved $d(\Delta R/R_0)/dE$ for 2L $MoSe_2$ at 5 T. There is a clear positive (negative) Zeeman splitting of IX_{2L} (X^A_{2s}) indicative of a positive (negative) g-factor.

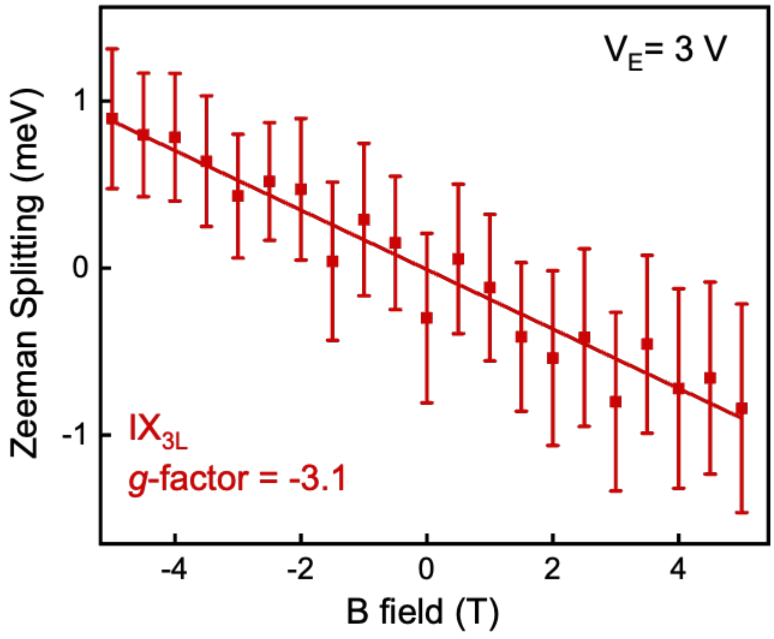

Fig. S10. **Zeeman splitting of IX_{3L} at V_E = 3 V.**

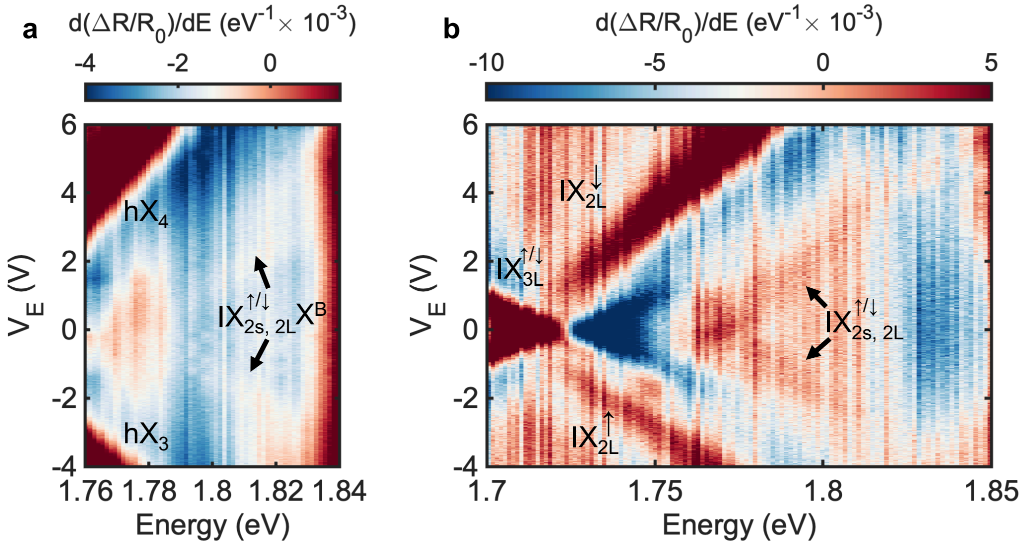

Fig. S11. **Excited state interlayer excitons in 2L and 3L $MoSe_2$ a,b** V_E dependence of the $d(\Delta R/R_0)/dE$ spectrum of 2L (a) and 3L $MoSe_2$ (b) in the main measurement locations shown the main text. The colour scale is saturated to better visualise the excited Rydberg state interlayer exciton ($IX_{2s,2L}$)

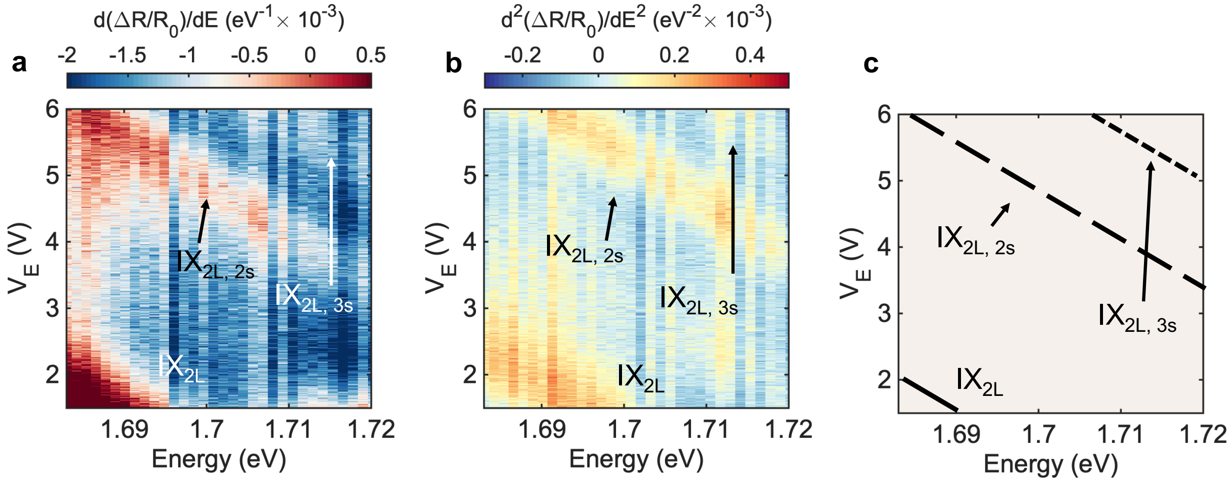

Fig. S12. **Ground and excited state interlayer excitons in 2L 2H-$MoSe_2$. a** The V_E dependence of the derivative with respect to energy of the $\Delta R/R_0$ signal ($d(\Delta R/R_0)/dE$) in the second location of 2L 2H-$MoSe_2$. **b** The second derivative ($d^2(\Delta R/R_0)/dE^2$) of the $d(\Delta R/R_0)/dE$) in panel (a). **c** Schematic image with guidelines to indicate the IX_{2L}, $IX_{2L,2s}$and $IX_{2L,3s}$, as seen experimentally in panels (a) and (b).

2. SUPPLEMENTAL TEXT AND FIGURES

A. S1: Phenomenological modelling of the hybridised exciton energies

1. Zero magnetic field ($B_z = 0$)

In order to capture the hybridisation-induced renormalisation of the different exciton resonance energies with increasing electric field, we employ a phenomenological model in which the hybridisation between different exciton states is treated as a coupling between oscillators with resonance energies corresponding to the exciton states being hybridised. According to this model, the electric-field-dependent energies ($E_{hyb,n}(V_E)$) and wave functions of the hybridised excitons ($|\Psi_{hyb,n}(V_E)\rangle$) can be calculated as

$$H_{hyb}(V_E)\,|\Psi_{hyb,n}(V_E)\rangle = E_{hyb,n}(V_E)\,|\Psi_{hyb,n}(V_E)\rangle\,, \tag{1}$$

where $H_{hyb}(V_E)$ is the Hamiltonian describing the hybrid exciton states, which can be written as $H_{hyb}(V_E) = H_0(V_E) + H_c$, with $H_0(V_E)$ and H_c being the Hamiltonians describing the bare exciton states and the coupling between the different exciton states, respectively. In this way, $H_0(V_E)$ can be calculated as

$$H_0(V_E) = \sum_{i,\sigma,\lambda} E_i(V_E)\left|X_i^{\sigma,\lambda}\right\rangle\left\langle X_i^{\sigma,\lambda}\right|, \tag{2}$$

where $E_i(V_E)$ denotes the energy of the bare exciton state $|X_i\rangle$ (i.e., $i = \mathrm{X}_{1s}^A, \mathrm{X}_{2s}^A, \mathrm{IX}_{2L}$ and IX_{3L}) at V_E, $\sigma = \Uparrow$ ($\Downarrow$) represents the spin up (down) configuration of the hole in the corresponding exciton state, and λ accounts for the layer degree of freedom (i.e., $\lambda = L_1, L_2$ or L_3). In order to reproduce the experimental results, we assume that the energies of the exciton resonances with intralayer nature (i.e., X_{1s}^A and X_{2s}^A excitons) are independent of the applied electric field, and V_E-dependent energies for excitons with interlayer character, for which we assume a linear Stark shift with the permanent dipole moments extracted from the experimental data. In the case of the coupling Hamiltonian, this needs to be calculated differently depending on the hybridisation mechanism. For exciton states that hybridise through direct spin-conserving, interlayer hole tunneling between the valence bands involved in each exciton (as it is the case for IX_{3L} and X_{1s}^A), the coupling mechanism can be treated as a layer-dependent, spin-conserving coupling Hamiltonian:

$$H_c = \sum_{i,j}\sum_{\sigma}\sum_{\lambda,\lambda'} \delta_{i\neq j}\delta_{\lambda\neq\lambda'}\kappa_{ij}\left(\left|X_i^{\sigma,\lambda}\right\rangle\left\langle X_j^{\sigma,\lambda'}\right| + \left|X_j^{\sigma,\lambda'}\right\rangle\left\langle X_i^{\sigma,\lambda}\right|\right). \tag{3}$$

On the contrary, the second-order effective coupling between IX_{2L} and the intralayer X_{2s}^A exciton facilitated via the A and B exciton admixture needs to be treated differently. As sketched in Fig. 2c in the main text, the proposed hybridisation mechanism between IX_{2L} and X_{2s}^A involves interlayer hole tunneling between the valence bands of IX_{2L} and the intralyer X^B exciton, which presents an intravalley admixture with the X^A exciton (and its excited states) [1–3]. Therefore, the coupling mechanism between IX_{2L} and X_{2s}^A can be treated as an effective interlayer, layer-selective, spin-flipping hole tunneling between the involved exciton states, which leads to the following coupling Hamiltonian:

$$H_c = \sum_{i,j}\sum_{\sigma\sigma'}\sum_{\lambda,\lambda'} \delta_{i\neq j}\delta_{\lambda\neq\lambda'}\delta_{\sigma\neq\sigma'}\kappa_{ij}\left(\left|X_i^{\sigma,\lambda}\right\rangle\left\langle X_j^{\sigma',\lambda'}\right| + \left|X_j^{\sigma',\lambda'}\right\rangle\left\langle X_i^{\sigma,\lambda}\right|\right), \tag{4}$$

where we have assumed in both Eq. (3) and Eq. (4) that the coupling constants κ_{ij} are independent of the applied electric field. As an example, below we show the Hamiltonian corresponding to the calculated hybridisation between IX_{2L} and X_{2s} shown in Fig. 2b of the main manuscript:

$$H_{hyb}^{IX_{2L}-X_{2s}}(V_E) = \begin{pmatrix} E_{2s}^{\Downarrow,L_2} & 0 & 0 & 0 & 0 & 0 & 0 & \kappa_{IX_{2L}-X_{2s}} \\ 0 & E_{2s}^{\Uparrow,L_2} & 0 & 0 & 0 & 0 & \kappa_{IX_{2L}-X_{2s}} & 0 \\ 0 & 0 & E_{2s}^{\Downarrow,L_1} & 0 & 0 & \kappa_{IX_{2L}-X_{2s}} & 0 & 0 \\ 0 & 0 & 0 & E_{2s}^{\Uparrow,L_1} & \kappa_{IX_{2L}-X_{2s}} & 0 & 0 & 0 \\ 0 & 0 & 0 & \kappa_{IX_{2L}-X_{2s}} & E_{2L}^{\Downarrow,L_2}(V_E) & 0 & 0 & 0 \\ 0 & 0 & \kappa_{IX_{2L}-X_{2s}} & 0 & 0 & E_{2L}^{\Uparrow,L_2}(V_E) & 0 & 0 \\ 0 & \kappa_{IX_{2L}-X_{2s}} & 0 & 0 & 0 & 0 & E_{2L}^{\Downarrow,L_1}(V_E) & 0 \\ \kappa_{IX_{2L}-X_{2s}} & 0 & 0 & 0 & 0 & 0 & 0 & E_{2L}^{\Uparrow,L_1}(V_E) \end{pmatrix},$$

where

$$E_{2s}^{\Downarrow,L_2} = E_{2s}^{\Uparrow,L_2} = E_{2s}^{\Downarrow,L_1} = E_{2s}^{\Uparrow,L_1} = 1.7433 \text{ eV}, \tag{5}$$

$$E_{2L}^{\Uparrow,L_1}(V_E) = E_{2L}^{\Downarrow,L_1}(V_E) = 1.7122 \text{ eV} + fV_E, \tag{6}$$

and

$$E_{2L}^{\Uparrow,L_2}(V_E) = E_{2L}^{\Downarrow,L_2}(V_E) = 1.7122 \text{ eV} - fV_E, \tag{7}$$

with f being the corresponding dipole moment expressed with the appropriate units (as extracted from fits to the experimental data). By diagonalising the Hamiltonian $H_{hyb}^{IX_{2L}-X_{2s}}(V_E)$ shown above, we find the V_E-dependent energies of the hybridised exciton states shown in Fig. 2b in the main manuscript.

2. Non-zero magnetic field ($B_z \neq 0$)

We also explore theoretically the hybridisation between the intralayer and interlayer exciton states when a magnetic field with a magnitude B_z is applied along the direction perpendicular to the sample interface (Faraday geometry). The model previously described can be adapted by including the effects that the applied magnetic field has on the energy of each bare exciton state depending on the orientation of its hole spin and layer degrees of freedom, which are intrinsically locked to the corresponding valley index ($\pm K$) [4]. As an example, the Hamiltonian shown above for the hybridisation between IX_{2L} and X_{2s} can be generalised to include the effect of an applied magnetic field ($H_{hyb}^{IX_{2L}-X_{2s}}(V_E, B_z)$) in both the bare and resulting hybrid exciton states by re-expressing the energies of the bare exciton states as follows:

$$E_{2s}^{\Downarrow,L_1} = E_{2s}^{\Uparrow,L_2} = 1.7433 \text{ eV } + g_{X_{2s}}\mu_B B_z/2, \tag{8}$$

$$E_{2s}^{\Uparrow,L_1} = E_{2s}^{\Downarrow,L_2} = 1.7433 \text{ eV } - g_{X_{2s}}\mu_B B_z/2, \tag{9}$$

$$E_{2L}^{\Uparrow(\Downarrow),L_1}(V_E) = 1.7122 \text{ eV} + fV_E \mp g_{IX_{2L}}\mu_B B_z/2, \tag{10}$$

and

$$E_{2L}^{\Uparrow(\Downarrow),L_2}(V_E) = 1.7122 \text{ eV} - fV_E \pm g_{IX_{2L}}\mu_B B_z/2, \tag{11}$$

with $g_{X_{2s}}$ ($g_{IX_{2L}}$) the g factor of the X_{2s}^A (IX_{2L}) exciton state and μ_B the Bohr magneton.

3. V_E-dependent g-factors of the hybridised excitons

Next, we employ the same phenomenological model to calculate the V_E-dependent change of the g-factors of the hybridised exciton states. Each hybridised exciton state $|\Psi_{hyb,n}\rangle$ can be written as a superposition of bare exciton states $|X_i\rangle$ according to

$$|\Psi_{hyb,n}(V_E)\rangle = \sum_{i,\sigma\lambda} C_i^{\sigma,\lambda}(V_E) \left| X_i^{\sigma,\lambda} \right\rangle, \tag{12}$$

where $C_i^{\sigma,\lambda}(V_E)$ are the electric-field-dependent complex amplitudes of the form $C_i^{\sigma,\lambda}(V_E) = \left\langle X_i^{\sigma,\lambda} \middle| \Psi_{hyb,n}(V_E) \right\rangle$, and $\sum_{i,\sigma,\lambda} |C_i^{\sigma,\lambda}(V_E)|^2 = 1$. Therefore, the V_E-dependent g-factor of the hybridised exciton states can be calculated as

$$g_{hyb,n}(V_E) = \sum_{i,\sigma,\lambda} |C_i^{\sigma,\lambda}(V_E)|^2 g_i, \tag{13}$$

where g_i represents the g-factor of the bare $|X_i\rangle$ states.

B. S2: Computational details

The atomic structures, the quasi-particle band structures and optical spectra have been obtained from DFT calculations using the VASP package [5, 6]. The projector augmented wave scheme [7, 8] has been used to to treat core electrons. We have set the lattice parameter value of 3.32 Å for all the runs. A grid of 21×21×1 k-points has been used, in conjunction with a vacuum height of 21.9 Å, for all the calculation cells. The geometry's optimization process has been performed at the PBE-D3 level [9] in order to include van der Waals interaction between layers. All the atoms were allowed to relax with a force convergence criterion below 0.005 eV/Å. Heyd-Scuseria-Ernzerhof (HSE) hybrid functional [10–12] has been used as approximation of the exchange-correlation electronic term, including SOC, to determine eigenvalues and wave functions as input for the full-frequency-dependent GW calculations [13] performed at the G_0W_0 level. The application of the electric field is done at this step, just before GW calculation process, considering that at small/moderate electric field values, its application is only a small perturbation of the band structures. An energy cutoff of 400 eV and a Gaussian smearing of 0.05 eV width have been chosen for partial occupancies, when a tight electronic minimization tolerance of 10^{-8} eV was set to determine with a good precision the corresponding derivative of the orbitals with respect to k needed in quasi-particle band structure calculations. The total number of states included in the GW procedure is set to 600, in conjunction with an energy cutoff of 100 eV for the response function, after a careful check of the direct band gap convergence (smaller than 0.1 eV as a function of k-points sampling). Band structures have been obtained after a Wannier interpolation procedure performed by the WANNIER90 program [14]. All optical excitonic transitions have been calculated by solving the Bethe-Salpeter Equation [15, 16], using the twelve highest valence bands and the sixteen lowest conduction bands to obtain eigenvalues and oscillator strengths on all systems. From these calculations, we report the absorbance values by using the imaginary part of the complex dielectric function. The calculated exciton admixtures are summarised in Table 1.

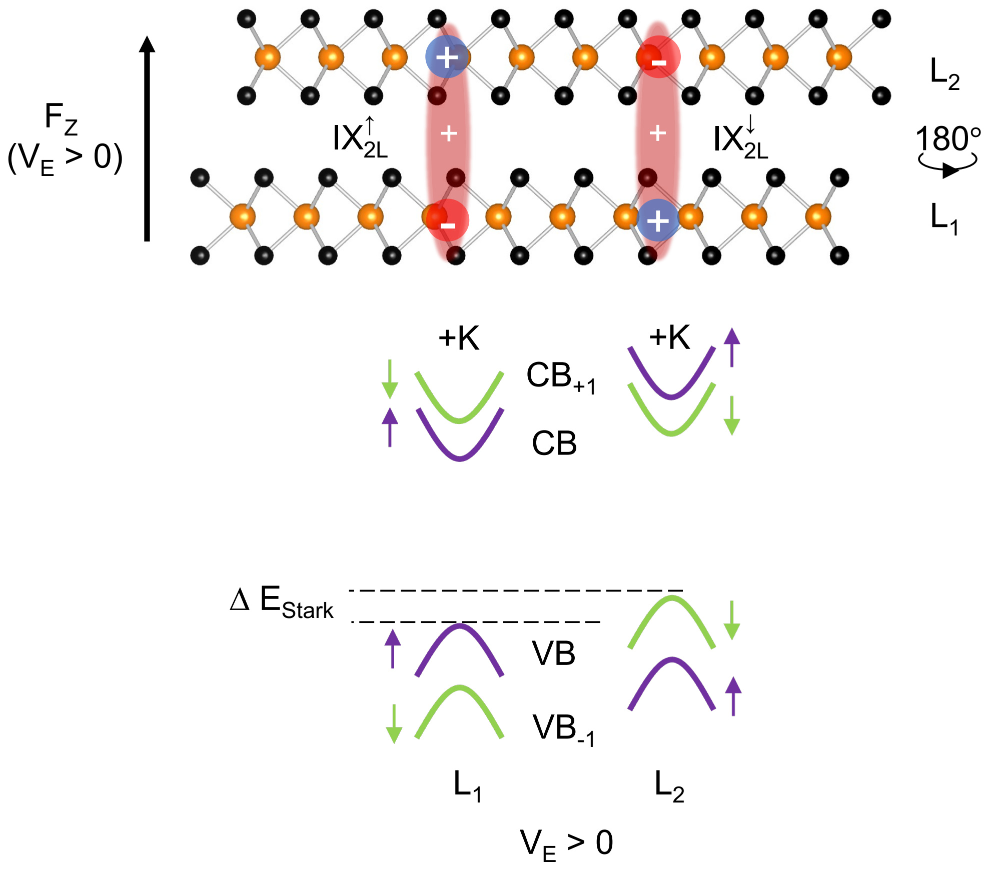

Fig. S13. **Scheme of the band structure in the +K valley under application of an electric field** The spin-orbit split upper and lower conduction (valence) bands of the +K valleys in L$_1$ and L$_2$ are labeled as $CB_{+1}^{L_1/L_2}$ (VB^{L_1/L_2}) and CB^{L_1/L_2} ($VB_{-1}^{L_1/L_2}$) respectively, as referred to by Table 1.

Supplementary Table

Exciton transition admixture (%) in 2L 2H-$MoSe_2$ without electric field								
	$VB^{L_2} \rightarrow CB^{L_2}$	$VB^{L_2}_{-1} \rightarrow CB^{L_2}_{+1}$	$VB^{L_1} \rightarrow CB^{L_1}$	$VB^{L_1}_{-1} \rightarrow CB^{L_1}_{+1}$	$VB^{L_2}_{-1} \rightarrow CB^{L_1}$	$VB^{L_2} \rightarrow CB^{L_1}_{+1}$	$VB^{L_1} \rightarrow CB^{L_2}_{+1}$	$VB^{L_1}_{-1} \rightarrow CB^{L_2}$
X^{A,L_2}_{1s}	94	2	0	0	0	0	0	4
X^{A,L_1}_{1s}	0	0	94	2	4	0	0	0
$IX^{\downarrow}_{2L}$	4	7	0	0	0	0	89	0
$IX^{\uparrow}_{2L}$	0	0	4	7	89	0	0	0
X^{A,L_2}_{2s}	84	5	0	7	0	0	0	4
X^{A,L_1}_{2s}	0	0	84	5	4	7	0	0
$IX^{\downarrow}_{2s,2L}$	4	31	0	0	0	0	65	0
$IX^{\uparrow}_{2s,2L}$	0	0	4	31	0	65	0	0
X^{B,L_2}	9	53	0	0	0	0	38	0
X^{B,L_1}	0	0	9	53	0	38	0	0

Exciton transition admixture (%) in 2L 2H-$MoSe_2$ with an electric field of 0.03 V/Å								
	$VB^{L_2} \rightarrow CB^{L_2}$	$VB^{L_2}_{-1} \rightarrow CB^{L_2}_{+1}$	$VB^{L_1} \rightarrow CB^{L_1}$	$VB^{L_1}_{-1} \rightarrow CB^{L_1}_{+1}$	$VB^{L_2}_{-1} \rightarrow CB^{L_1}$	$VB^{L_2} \rightarrow CB^{L_1}_{+1}$	$VB^{L_1} \rightarrow CB^{L_2}_{+1}$	$VB^{L_1}_{-1} \rightarrow CB^{L_2}$
X^{A,L_2}_{1s}	92	3	0	0	0	0	1	4
X^{A,L_1}_{1s}	0	0	90	2	4	4	0	0
$IX^{\downarrow}_{2L}$	30	7	0	0	0	0	62	1
$IX^{\uparrow}_{2L}$	0	0	5	5	90	0	0	0
X^{A,L_2}_{2s}	52	9	0	0	0	0	38	1
X^{A,L_1}_{2s}	0	0	64	12	8	16	0	0
$IX^{\downarrow}_{2s,2L}$	3	54	0	0	0	0	43	0
$IX^{\uparrow}_{2s,2L}$	0	0	30	20	5	45	0	0
X^{B,L_2}	0	46	0	1	0	0	53	0
X^{B,L_1}	0	0	2	94	4	0	0	0

TABLE I. Supplementary tables of the exciton transition admixture in 2L 2H-$MoSe_2$ at 0 (upper table) and 0.03 V/Å (lower table) electric field, obtained from *GW*-BSE calculations.

C. S3: Effects of an applied magnetic field on the X_{2s}^{A}-IX_{2L} hybridisation in 2L $MoSe_2$

The application of a vertical magnetic field (Faraday configuration) leads to a different evolution of the σ^+ and σ^--polarised hybrid excitons. Supplementary Fig. S14a shows the experimental V_E-dependent evolution of the hybrid IX_{2L}-X_{2s} exciton energies in 2L $MoSe_2$ in the same sample spot discussed in the main manuscript for σ^-- and σ^+-resolved spectroscopy under an applied magnetic field of 5 T (left and right panels, respectively). The horizontal solid and dashed lines indicate the V_E at which we observe the highest IX_{2L}-X_{2s} hybridisation for each helicity-resolved measurement, which show that the energy anti-crossing of the IX_{2L} and X_{2s} exciton features occurs at lower V_E for the σ^+-polarised exciton branches. This experimental observation agrees well with the predictions of our phenomenological coupling model. Supplementary Fig. S14b shows the calculated V_E-dependent evolution of the hybrid IX_{2L}-X_{2s} exciton energies in 2L with σ^+ (solid lines) and σ^--polarised (dashed lines) optical transitions for an applied magnetic field of 5 T. In the calculation we employed the parameters obtained from the fit to the experimental data at $B_z = 0$ T shown in Fig. 2b in the main text, and included the g factors of the different bare exciton states estimated from the analysis shown in Fig. 4. As shown in these results, our phenomenological model captures well the B_z-induced shift of the V_E at which the IX_{2L}-X_{2s} hybrid excitons present the highest degree of hybridisation depending on their spin-valley-layer degree of freedom.

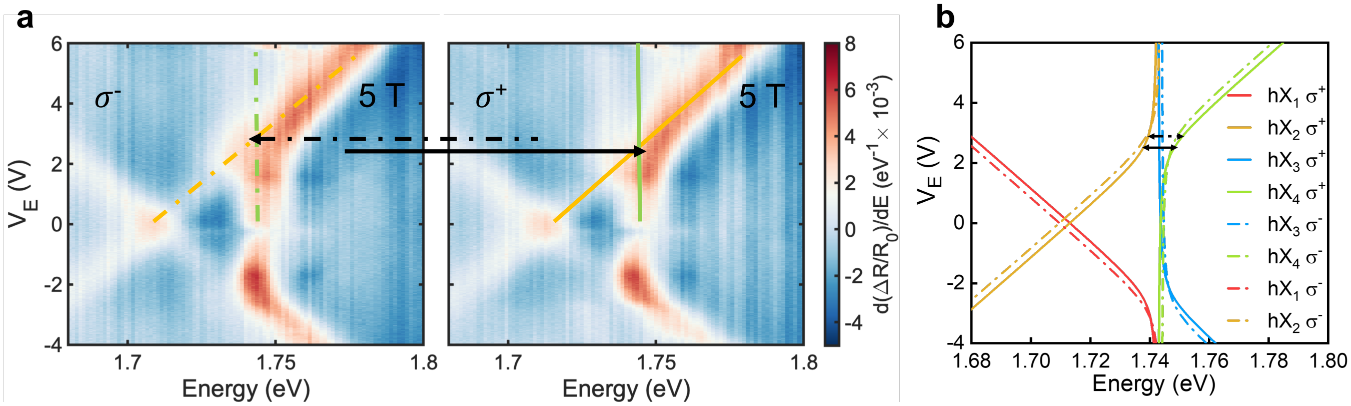

Fig. S14. **Tuning the IX_{2L}-X_{2s} hybridisation with an applied magnetic field a** V_E dependent σ^- (left panel) and σ^+ (right panel) resolved $d(\Delta R/R_0)/dE$ for an applied magnetic field of 5 T in 2L $MoSe_2$. The solid (dashed) black line indicates the V_E at which the IX_{2L}-X_{2s} hybrid excitons present the highest degree of hybridisation for σ^+ (σ^-). The coloured lines represent guidelines for the V_E dependent dispersions of IX_{2L} (yellow) and X_{2s} (green), and the V_E at which they would intersect (in the absence of hybridisation). **b** Calculated V_E-dependent evolution of the hybrid IX_{2L}-X_{2s} exciton energies in 2L $MoSe_2$ with σ^+ (solid lines) and σ^--polarised (dashed lines) optical transitions for an applied magnetic field of 5 T.

D. S4: Experimental signatures of X_{1s}^{A}-IX_{2L} hybridisation in 3L $MoSe_2$

As discussed in the main text, there is a reduced energy splitting between $X_{1s}^{A,L_1(L_3)}$ and IX_{2L} in our 3L $MoSe_2$ compared to the 2L. As a consequence, we are able to observe experimental signatures of the coupling between these two exciton species at $|V_E| \sim 6$ V (see Suppl. Figs. S15a and S15b), which corroborate our results for the 2L 2H-$MoSe_2$. Such a hybridisation between $X_{A,1s}^{L_1(L_3)}$ and IX_{2L} in our 3L $MoSe_2$ is also well captured in our simulations. Figure S16 shows the calculated $d(\Delta R/R_0)/dE$ spectrum for our 3L $MoSe_2$ region, in which we assume that the $d(\Delta R/R_0)/dE$ optical response of each exciton state can be approximated by a Lorentzian function. The energy, linewidths, and relative oscillator strengths of the different exciton states at $V_E = 0$ V have been chosen to reproduce the experimental plots in Suppl. Figs. S15a and S15b, while their evolution with V_E is naturally taken into account by our phenomenological coupling model. For completeness, using the layer selection rules discussed in S3 and section B, considering the exciton configuration in 3L $MoSe_2$ (see Fig. 1 in the main text) including the three different X_{1s}^{A} excitons (i.e. one in each layer), two IX_{3L} with opposite polarities ($IX_{3L}^{\uparrow(\downarrow)}$), three X_{2s}^{A} excitons ($X_{2s}^{A,L_1(L_3)}$ and X_{2s}^{A,L_2}), four IX_{2L} with different polarities and layer configurations, $IX_{3L}^{\uparrow(\downarrow)}$, and $IX_{3L}^{*\uparrow(\downarrow)}$ with same dipole moment as ground state. We assume there are spin-conserved couplings between $IX_{3L}^{\uparrow(\downarrow)}$ and $X_{2s}^{A,L_3(L_1)}$ and between $IX_{3L}^{\uparrow(\downarrow)}$ and $X_{1s}^{A,L_1(L_3)}$. Note that X_{1s}^{A,L_2} and X_{2s}^{A,L_2} couple with both $IX_{2L}^{\downarrow}$ (transition between L_1 and L_2) and $IX_{2L}^{\uparrow}$ (transition between L_2

and L_3). The coupling constant and spin- and layer-selection rule for $IX_{2s,3L}^{\uparrow(\downarrow)}$ are set the same as $IX_{3L}^{\uparrow(\downarrow)}$. We also include couplings between $IX_{2L}^{\uparrow(\downarrow)}$ and $X_{1s}^{A,L_1(L_3)}$ and $X_{2s}^{A,L_1(L_3)}$. We set the $\kappa_{2L-2s} = \kappa_{2L-1s} = 2$ meV and $\kappa_{3L-2s} = \kappa_{3L-1s} = 4$ meV. Suppl. Fig. S17 shows the calculated absorption spectrum for the alternate measurement location of the 3L region (see Fig. 5 in the main text for the experimental data).

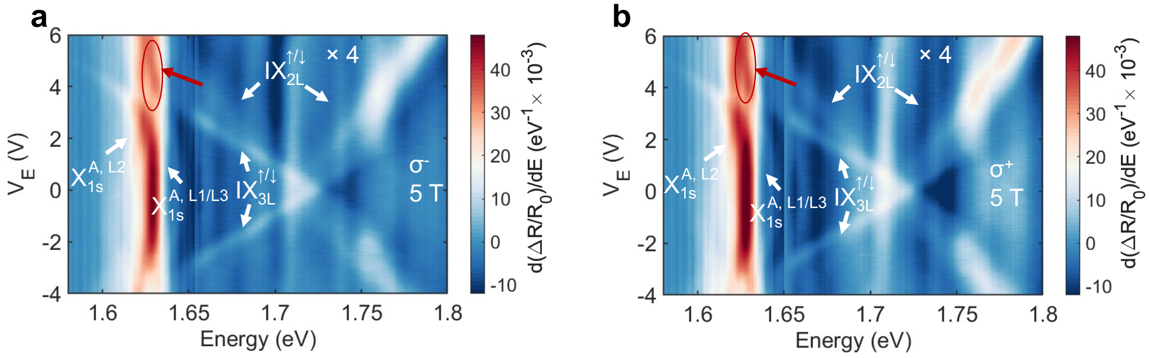

Fig. S15. **Signatures of X_{1s}^{A}-IX_{2L} hybridisation in 3L $MoSe_2$ a,b** V_E dependent σ^- (a) and σ^+ (b) resolved $d(\Delta R/R_0)/dE$ for an applied magnetic field of 5 T in 3L $MoSe_2$ where $X_{1s}^{A,L1/L3}$, $X_{1s}^{A,L2}$, IX_{3L}, IX_{2L} are labelled. The red arrows indicate hybridisation between IX_{2L} and $X_{1s}^{A,L1/L3}$ which can be observed in the 3L $MoSe_2$ due to the reduced energy spacing between $X_{1s}^{A,L1/L3}$ and IX_{2L} in comparison to the 2L $MoSe_2$.

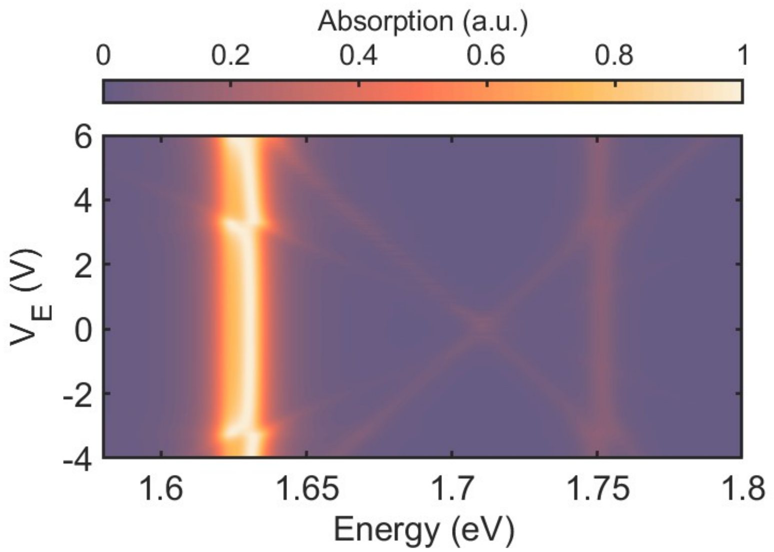

Fig. S16. **Simulated absorption spectra of 3L $MoSe_2$** Calculated $d(\Delta R/R_0)/dE$ spectrum for our 3L $MoSe_2$ region using a phenomenological coupling model, in which we assume that the $d(\Delta R/R_0)/dE$ optical response of each exciton state can be approximated by a Lorentzian function. The energy, linewidths, and relative oscillator strengths of the different exciton states at $V_E = 0$ V have been chosen to reproduce the experimental plots in Suppl. Figs. S15a and S15b, while their evolution with V_E is naturally taken into account by our phenomenological coupling model.

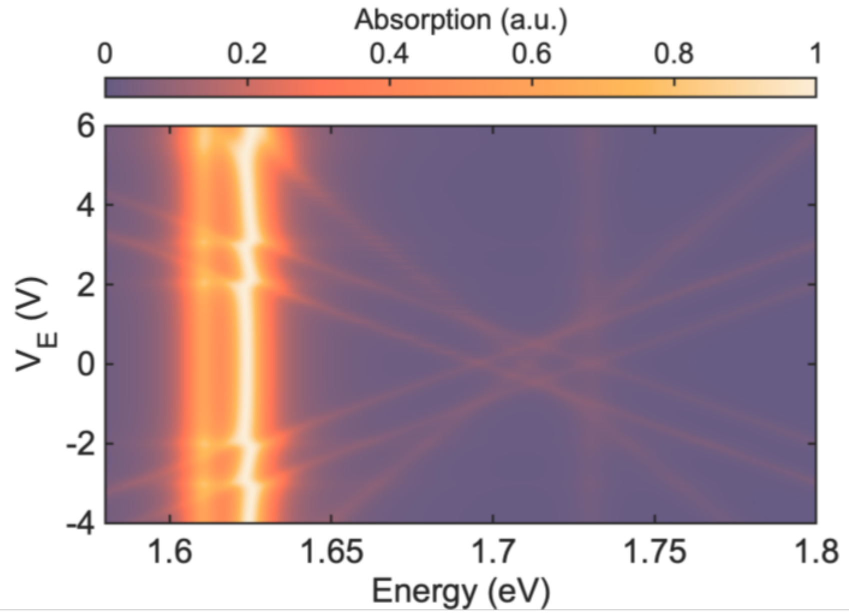

Fig. S17. **Simulated absorption spectra for the alternate location of 3L $MoSe_2$.** Calculated $d(\Delta R/R_0)/dE$ spectrum for the alternate 3L $MoSe_2$ region using a phenomenological coupling model, in which we assume that the $d(\Delta R/R_0)/dE$ optical response of each exciton state can be approximated by a Lorentzian function. The energy, linewidths, and relative oscillator strengths of the different exciton states at $V_E = 0$ V have been chosen to reproduce the experimental plots in Fig. 5b, considering the IX^{*}_{3L} which also couples with X_A. Their evolution with V_E is naturally taken into account by our phenomenological coupling model.

E. S5: Summary of hybrid IX in multilayer TMD system

First in this manuscript we show that for the hybrid IX_{2L} in 2H-2L $MoSe_2$, the up (down) IX dipole couples with X^A_{2s} exciton in L_1 (L_2) through second ordering coupling. In previous works, the focus was on the IX_{2L} coupling with B exciton through spin conserved hole tunnelling (first order coupling). For the hybrid IX_{3L} in 2H-3L $MoSe_2$ or R-stacked $MoSe_2$/BN/$MoSe_2$, it couples with the intralayer A exciton in L_1 and L_3 through spin conserved hole tunnelling (first order coupling) while coupling with L_2 is spin forbidden. Similarly in a parallel multilayer 2H-WSe_2 work [17], the dipolar IX_{3L} couples with each odd number layers like L_1, L_3, L_5 etc., which forms the layer selective spin valley locked superlattice.

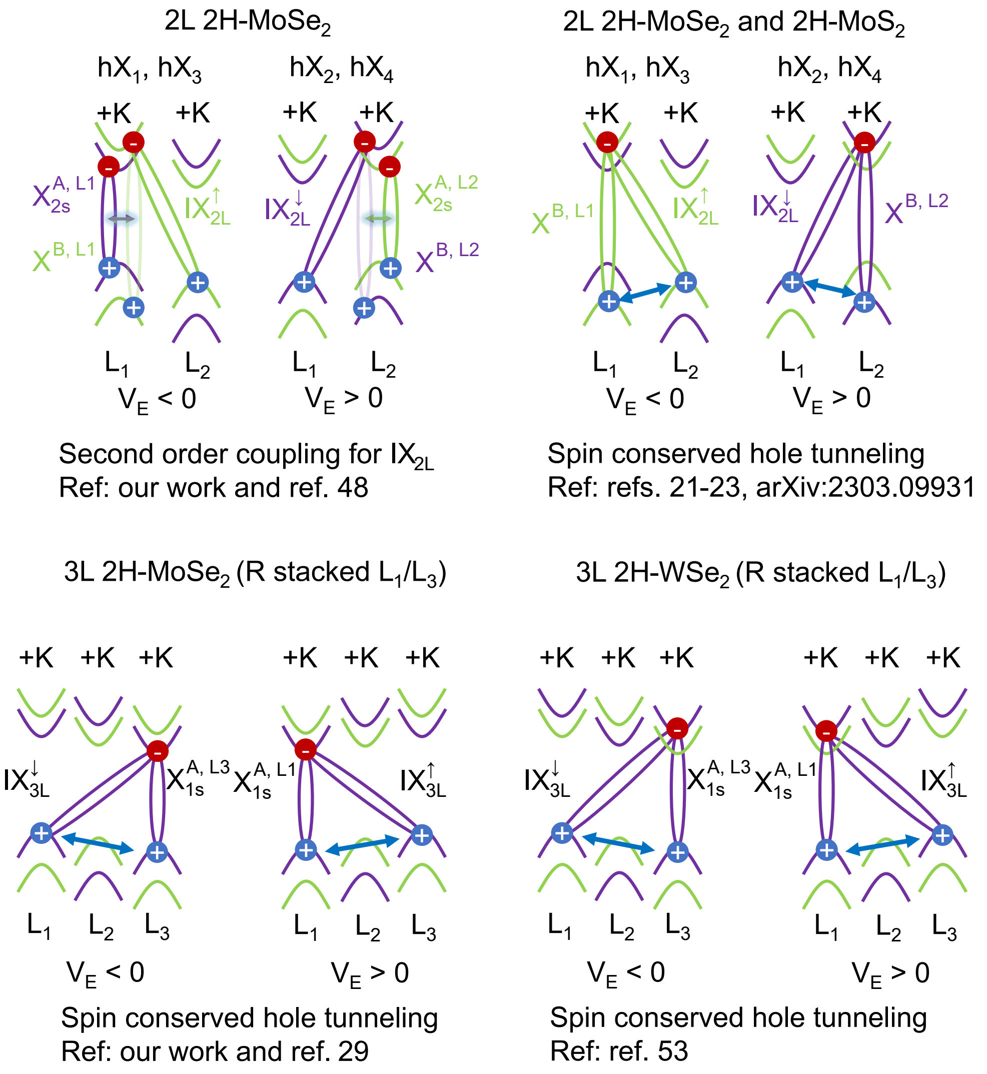

Fig. S18. **Summary of different types of intra/interayer exciton hybridization in TMD system.**

F. S6. Discussion about the energetic order of IX_{2L} and IX_{3L}

The absorption energy of the excitons is determined by the interband transition (Fig. 1) and binding energies. For IX_{2L} (IX_{3L}), in the bare exciton picture, the interband transition is from the upper (lower) conduction band, therefore the IX_{2L} has a larger interband energy than IX_{3L} (estimated to be $\approx$ 10's of meV [18–20]). For the binding energy which is based on the effective electron-hole separation, the IX_{2L} has a larger Coulomb binding energy than IX_{3L}, i.e. in principle IX_{2L} also has larger energy reduction from the interband energy (see Fig. S19 for a summary). However, the binding energies of both IX_{2L} and IX_{3L} are subject to their local dielectric environments due to their large spatial wavefunctions[21, 22], as well as common TMD sample inhomogeneity from local strain. Finally, we note once again that the cartoon-like 'bare' exciton picture is a simplification. Combining these factors, it is clear there is a delicate balance that can lead to disparity in the precise energies and even relative ordering between IX_{2L} and IX_{3L}.

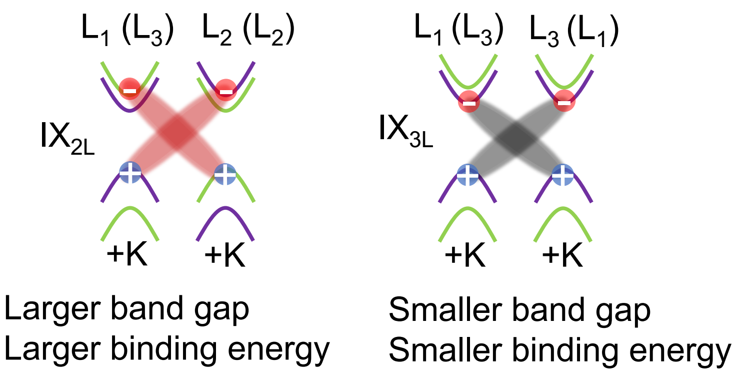

Fig. S19. **Schematic image of the interband transition of IX_{2L} and IX_{3L} in 2L and 3L $MoSe_2$, respectively**

G. S7. Additional charaterization of excited state IXs

Here we demonstrate the doping dependent ($V_D = V_T = V_B$) dispersion of the $IX_{2s,2L}$, which shows a drastic decay compared to the ground state counterpart and even X^A_{2s}. This is a typical feature of excited state excitons, as the enlarged spatial wave function leads to smaller k space extension such that phase space filling happens much faster with moderate doping.

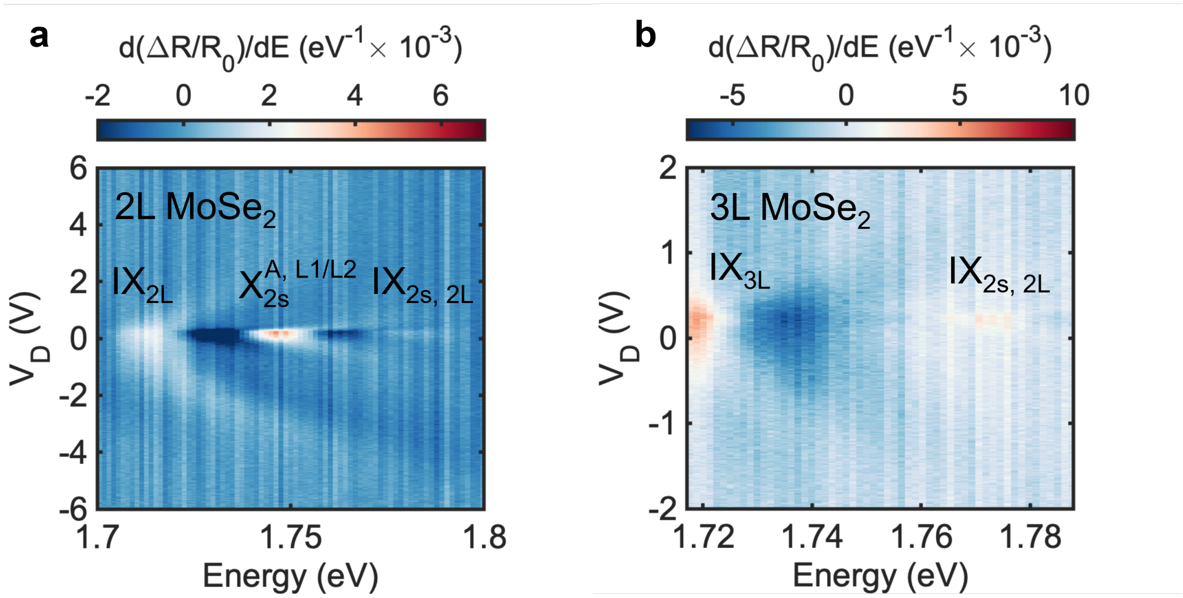

Fig. S20. **$d(\Delta R/R_0)/dE$ spectra of 2L and 3L $MoSe_2$ as function of doping** (a) V_D dependent $d(\Delta R/R_0)/dE$ spectra of 2L $MoSe_2$. (b) V_D dependent $d(\Delta R/R_0)/dE$ spectra of 3L $MoSe_2$.

We also investigated magnetic field dependent reflectance of 2L $MoSe_2$ and compared the valley exciton dispersion. As we sweep the field from -5 to 5 T, in σ^- polarization, the IX_{2L} (X^A_{2s}) loses (gains) oscillator strength, and vice versa for σ^+ polarization. This is in line with our interpretation as IX_{2L} (X^A_{2s}) has positive (negative) bare exciton g-factor. Importantly we observed similar trends for $IX_{2s,2L}$ and IX_{2L}. This is a strong indication that both transitions have positive g-factors and an interlayer nature. We also notice the diamagnetic shift of $IX_{2s,2L}$, is larger than the IX_{2L} diamagnetic shift, which is a typical behavior of 2s excited states. Similar diamagnetic shift trends have been reported for X^A_{2s} in 1L $MoSe_2$ [23].

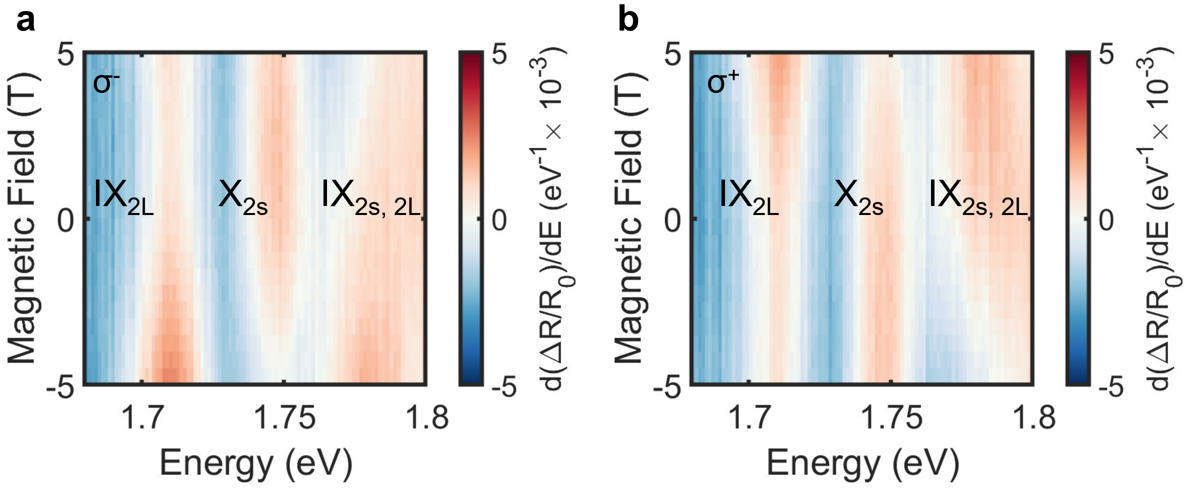

Fig. S21. **Magnetic field dependent reflectance of 2L $MoSe_2$ a,b** Magnetic field dependent σ^- (a) and σ^+ (b) resolved $\mathrm{d}(\Delta R/R_0)/\mathrm{d}E$ of 2L $MoSe_2$.

In addition, similar to $\mathrm{IX}_{3s,2L}$ in Fig. S9, here we show that we can also resolve a vanishing higher order IX_{3L}: two parallel IX (next to the ground state IX) are visible in the electric field sweep of the reflectance data shown below.

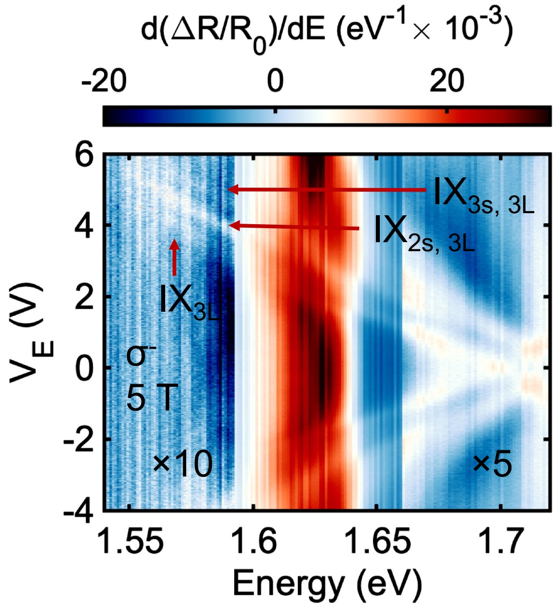

Fig. S22. **Ground and excited state interlayer excitons in 3L $MoSe_2$.** The parralleled IX_{3L}, $\mathrm{IX}_{2s,3L}$ and $\mathrm{IX}_{3s,3L}$ are labeled.

H. S8. Rydberg binding energies of excited state IXs

Finally, we extract the energies of 1s, 2s, and 3s of IX_{2L} to fit with the 2D hydrogen model to further understand the binding energy [24]. The experimental data can be partially fit to the 2D hydrogen model, which is encouraging for following theoretical investigation and dielectric engineering experiments.

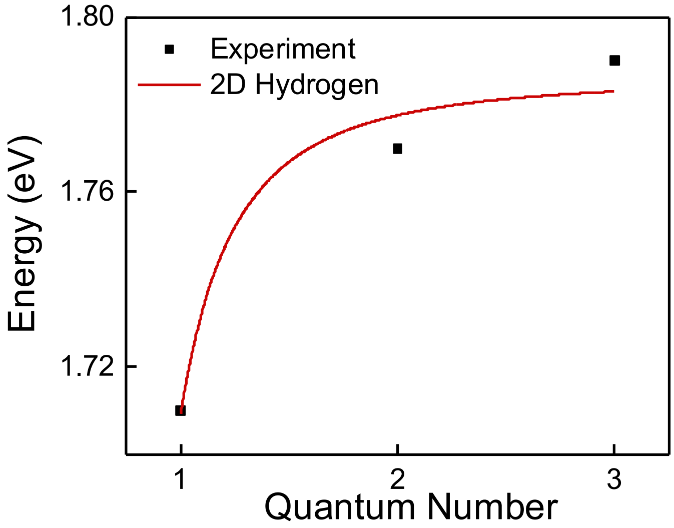

Fig. S23. **Extracted energy of ground and excited state IX_{2L} and fit based on 2D hydrogen model.**

---

[1] Nadine Leisgang, Shivangi Shree, Ioannis Paradisanos, Lukas Sponfeldner, Cedric Robert, Delphine Lagarde, Andrea Balocchi, Kenji Watanabe, Takashi Taniguchi, Xavier Marie, *et al.*, "Giant stark splitting of an exciton in bilayer mos_2," Nat. Nanotecnol. **15**, 901–907 (2020).

[2] Lukas Sponfeldner, Nadine Leisgang, Shivangi Shree, Ioannis Paradisanos, Kenji Watanabe, Takashi Taniguchi, Cedric Robert, Delphine Lagarde, Andrea Balocchi, Xavier Marie, *et al.*, "Capacitively and inductively coupled excitons in bilayer mos_2," Phys. Rev. Lett. **129**, 107401 (2022).

[3] Joakim Hagel, Samuel Brem, and Ermin Malic, "Electrical tuning of moiré excitons in mose2 bilayers," 2D Mater. **10**, 014013 (2022).

[4] Aaron M Jones, Hongyi Yu, Jason S Ross, Philip Klement, Nirmal J Ghimire, Jiaqiang Yan, David G Mandrus, Wang Yao, and Xiaodong Xu, "Spin–layer locking effects in optical orientation of exciton spin in bilayer wse_2," Nat. Phys. **10**, 130–134 (2014).

[5] G. Kresse and J. Hafner, "*Ab initio* molecular dynamics for liquid metals," Phys. Rev. B **47**, 558–561 (1993).

[6] G. Kresse and J. Furthmüller, "Efficient iterative schemes for *ab initio* total-energy calculations using a plane-wave basis set," Phys. Rev. B **54**, 11169–11186 (1996).

[7] P E Blöchl, "Projector augmented-wave method," Phys. Rev. B **50**, 17953 (1994).

[8] G Kresse and D Joubert, "From ultrasoft pseudopotentials to the projector augmented-wave method," Phys. Rev. B **59**, 1758–1775 (1999).

[9] Stefan Grimme, Jens Antony, Stephan Ehrlich, and Helge Krieg, "A consistent and accurate ab initio parametrization of density functional dispersion correction (DFT-D) for the 94 elements H-Pu," J. Chem. Phys. **132**, 154104–19 (2010).

[10] J Heyd and G E Scuseria, "Assessment and validation of a screened Coulomb hybrid density functional," J. Chem. Phys. **120**, 7274 (2004).

[11] J Heyd, J E Peralta, G E Scuseria, and R L Martin, "Energy band gaps and lattice parameters evaluated with the Heyd-Scuseria-Ernzerhof screened hybrid functional," J. Chem. Phys. **123**, 174101 (2005).

[12] J Paier, M Marsman, K Hummer, G Kresse, I C Gerber, and J G Ángyán, "Screened hybrid density functionals applied to solids," J. Chem. Phys. **124**, 154709 (2006).

[13] M. Shishkin and G. Kresse, "Implementation and performance of the frequency-dependent gw method within the paw framework," Phys. Rev. B **74**, 035101 (2006).

[14] Arash A Mostofi, Jonathan R Yates, Young-Su Lee, Ivo Souza, David Vanderbilt, and Nicola Marzari, "wannier90: A tool for obtaining maximally-localised Wannier functions," Comput. Phys. Commun. **178**, 685–699 (2008).

[15] W Hanke and L J Sham, "Many-Particle Effects in the optical Excitations of a semiconductor," Phys. Rev. Lett. **43**, 387 (1979).

[16] M Rohlfing and S G Louie, "Electron-hole Excitations in Semiconductors and Insulators," Phys. Rev. Lett. **81**, 2312–2315 (1998).

[17] Yinong Zhang, Chengxin Xiao, Dmitry Ovchinnikov, Jiayi Zhu, Xi Wang, Takashi Taniguchi, Kenji Watanabe, Jiaqiang Yan, Wang Yao, and Xiaodong Xu, "Every-other-layer dipolar excitons in a spin-valley locked superlattice," Nat. Nanotechnol. **18**, 501–506 (2023).

[18] Gui-Bin Liu, Wen-Yu Shan, Yugui Yao, Wang Yao, and Di Xiao, "Three-band tight-binding model for monolayers of group-vib transition metal dichalcogenides," Phys. Rev. B **88**, 085433 (2013).

[19] K. Kośmider, J. W. González, and J. Fernández-Rossier, "Large spin splitting in the conduction band of transition metal dichalcogenide monolayers," Phys. Rev. B **88**, 245436 (2013).

[20] Zefang Wang, Liang Zhao, Kin Fai Mak, and Jie Shan, "Probing the spin-polarized electronic band structure in monolayer transition metal dichalcogenides by optical spectroscopy," Nano Lett. **17**, 740–746 (2017).

[21] Archana Raja, Andrey Chaves, Jaeeun Yu, Ghidewon Arefe, Heather M Hill, Albert F Rigosi, Timothy C Berkelbach, Philipp Nagler, Christian Schüller, Tobias Korn, *et al.*, "Coulomb engineering of the bandgap and excitons in two-dimensional materials," Nat. Commun. **8**, 15251 (2017).

[22] Philipp Steinleitner, Philipp Merkl, Alexander Graf, Philipp Nagler, Kenji Watanabe, Takashi Taniguchi, Jonas Zipfel, Christian Schuller, Tobias Korn, Alexey Chernikov, *et al.*, "Dielectric engineering of electronic correlations in a van der waals heterostructure," Nano Lett. **18**, 1402–1409 (2018).

[23] M Goryca, Jing Li, Andreas V Stier, Takashi Taniguchi, Kenji Watanabe, Emmanuel Courtade, Shivangi Shree, Cedric Robert, Bernhard Urbaszek, Xavier Marie, *et al.*, "Revealing exciton masses and dielectric properties of monolayer semiconductors with high magnetic fields," Nat. Commun. **10**, 4172 (2019).

[24] Alexey Chernikov, Timothy C Berkelbach, Heather M Hill, Albert Rigosi, Yilei Li, Burak Aslan, David R Reichman, Mark S Hybertsen, and Tony F Heinz, "Exciton binding energy and nonhydrogenic rydberg series in monolayer ws_2," Phys. Rev. Lett. **113**, 076802 (2014).
